# Geographical, Temporal and Environmental Determinants of Bryophyte Species Richness in the Macaronesian Islands

**DOI:** 10.1371/journal.pone.0101786

**Published:** 2014-07-08

**Authors:** Silvia C. Aranda, Rosalina Gabriel, Paulo A. V. Borges, Ana M. C. Santos, Eduardo Brito de Azevedo, Jairo Patiño, Joaquín Hortal, Jorge M. Lobo

**Affiliations:** 1 Departamento de Biogeografía y Cambio Global, Museo Nacional de Ciencias Naturales, Madrid, Spain; 2 Azorean Biodiversity Group and Platform for Enhancing Ecological Research & Sustainability, Departamento de Ciências Agrárias, Universidade dos Açores, Angra do Heroísmo, Açores, Portugal; 3 Centro de Biologia Ambiental and Platform for Enhancing Ecological Research & Sustainability, Faculdade de Ciências de Lisboa, Universidade de Lisboa, Lisbon, Portugal; 4 Centro do Clima, Meteorologia e Mudanças Globais, Departamento de Ciências Agrárias, Universidade dos Açores, Angra do Heroísmo, Açores, Portugal; 5 Institute of Botany, University of Liège, Liège, Belgium; 6 Departmento de Biología Vegetal, Universidad de La Laguna, Tenerife, Spain; Consiglio Nazionale delle Ricerche (CNR), Italy

## Abstract

Species richness on oceanic islands has been related to a series of ecological factors including island size and isolation (i.e. the Equilibrium Model of Island Biogeography, EMIB), habitat diversity, climate (i.e., temperature and precipitation) and more recently island ontogeny (i.e. the General Dynamic Model of oceanic island biogeography, GDM). Here we evaluate the relationship of these factors with the diversity of bryophytes in the Macaronesian region (Azores, Madeira, Canary Islands and Cape Verde). The predictive power of EMIB, habitat diversity, climate and the GDM on total bryophyte richness, as well as moss and liverwort richness (the two dominant bryophyte groups), was evaluated through ordinary least squares regressions. After choosing the best subset of variables using inference statistics, we used partial regression analyses to identify the independent and shared effects of each model. The variables included within each model were similar for mosses and liverworts, with orographic mist layer being one of the most important predictors of richness. Models combining climate with either the GDM or habitat diversity explained most of richness variation (up to 91%). There was a high portion of shared variance between all pairwise combinations of factors in mosses, while in liverworts around half of the variability in species richness was accounted for exclusively by climate. Our results suggest that the effects of climate and habitat are strong and prevalent in this region, while geographical factors have limited influence on Macaronesian bryophyte diversity. Although climate is of great importance for liverwort richness, in mosses its effect is similar to or, at least, indiscernible from the effect of habitat diversity and, strikingly, the effect of island ontogeny. These results indicate that for highly vagile taxa on oceanic islands, the dispersal process may be less important for successful colonization than the availability of suitable ecological conditions during the establishment phase.

## Introduction

The Equilibrium Model of Island Biogeography (EMIB) states that, other things being equal, area and geographic isolation are the two main factors determining extinction and immigration rates, which in turn regulate the level of species richness that is reached at a dynamic equilibrium [1], [2]. Although many hypotheses have been proposed to explain the role of area in species richness patterns [3], in its original formulation the EMIB postulated the effect of area *per se* referring specifically to demographic processes (i.e. smaller areas support smaller populations that are hence more prone to species extinctions). Despite its importance in the development of ecology and biogeography, the EMIB has been criticized for the lack of ability of its simple mechanisms to account for variations in species richness (e.g. [4]). In fact, models based on additional factors have been suggested to also account for island diversity, including energy [5], [6], habitat diversity [7] or island ontogeny in the particular case of oceanic archipelagos [8].

In essence, the models considering energy relate the amount of available resources with the possibility of maintaining higher population sizes and therefore more species [6]. The variety of resource types (e.g. habitat diversity) would also promote the coexistence of more species by diminishing interspecific competition and increasing sympatric speciation through ecological space partitioning (see [9]). The ontogenetic evolution of the island itself may affect its carrying capacity and hence species richness, because the variations in area and structural complexity occurring during the island's life cycle influence both typical immigration-extinction dynamics and diversification by *in situ* speciation (i.e. the General Dynamic Model of oceanic island biogeography or GDM) [8], [10]. The effects of energy, habitat diversity and island ontogeny on species richness have been typically examined using surrogates such as actual evapotranspiration or other climatic factors (e.g. [11]), topographic variables or habitat classifications (e.g. [12]), and the maximum geological age dated for islands (e.g. [13], [14]), respectively. Numerous studies have evaluated some of these models for a wide variety of taxa and archipelagos, either confirming or rejecting their predictions (e.g. [15]–[17]).

Although all these factors are known to affect island species richness, few attempts have been made to assess their comparative importance within a single evaluation (but see [11], [18], [19]). This may be due to the fact that most predictors are often correlated and therefore it is difficult to separate their true influence on species richness through common statistical techniques [20] (see also [21]). In addition, generalizations about the importance of the processes underlying these predictors depend on the idiosyncratic characteristics of both islands (e.g. the range of variation in area, isolation or elevation) and taxa (e.g. dispersal ability or life cycle). For example, the influence of isolation on immigration depends on the dispersal ability of the taxon, which in turn limits the probability of *in situ* speciation [14], [22], [23]. Similarly, the influence of environmental heterogeneity (habitat or climatic diversity) on the successful establishment of species varies according to their physiological and ecological tolerances (i.e. niche breadth), eventually determining the shape of richness–environment relationships (see [24]).

Bryophytes – which encompass hornworts, liverworts and mosses – are unique among land plants because: (i) the gametophyte is the dominant phase of the life cycle comprising the leafy or thalloid plants; and (ii) the sporophyte, which consists mainly of a short-lived small “capsule”, is always attached to and dependent on the gametophyte. Both singularities make the two generations of the life cycle to contribute significantly to the dispersal and establishment processes [25]. In addition, contrary to seed plants and ferns, they lack complex vascular tissues and developed a poikilohydric strategy that allows them to absorb water over their whole surface by capillarity, being able to remain metabolically inactive when dry conditions exist. Furthermore, bryophytes are characterized by extremely low levels of endemism in oceanic floras (see [26] for review], which is thought to be a consequence of the high dispersal ability of the group [27].

Despite these interesting features, bryophytes have received relatively little attention in island biogeography studies compared to other plant groups (but see [22], [28]–[30]). Most of these works include only one archipelago (but see [17], [22]) or do not consider all the above-mentioned factors, and in particular climate (but see [29]). Also, the effect of climate on large-scale species richness gradients has been occasionally analyzed in spore-dispersed plants [31], being mostly studied indirectly through its correlation with latitude and altitude (e.g. [32]–[34]). In the present study we examine the role of geographical, temporal and environmental factors on the between-island variation of bryophyte species richness in the Macaronesian Region (i.e. Azores, Madeira, Canary Islands and Cape Verde). Specifically, we evaluate four non-exclusive hypotheses under the following premises:

***H_1_*** **.** The Equilibrium Model of Island Biogeography (EMIB) should not significantly account for the variation in species richness of bryophytes, or its effect should be negligible. We expect that geographic isolation will not have a significant effect on immigration rates since bryophytes have the potential to disperse long distances by spores [35], [36]. In spite of some discrepancies [17], [22], [37], the dispersal ability of bryophytes should in turn limit the influence of area *per se* because the high rescue effect from surrounding source populations would minimize species extinctions.

***H_2_*** **.** The General Dynamic Model of oceanic island biogeography (GDM) should not be of high relevance for bryophytes because the effect of area in species richness should be minimized with increasing dispersal ability and also because former studies suggested that time *per se* appears to have little support in predicting species richness in the group [17].

***H_3_*** **.** Habitat diversity (HD) should have a significant effect on species richness because bryophyte communities are known to show significant degrees of compositional turnover between different habitats [29], [38]–[40].

***H_4_*** **.** Precipitation and temperature (CLIMATE) should have a strong effect on species richness, since sexual reproduction and photosynthesis in bryophytes are highly dependent on water availability, and optimal growth occurs with moderate temperatures [41].

## Results

Univariate regressions between dependent variables (*S_TOT_*, *S_M_* and *S_L_*) and all the considered predictors showed similar results for mosses and liverworts (Table 1). From the predictors representing the EMIB only area (*A*) was significantly related to moss species richness variation. In the case of GDM, both the linear and quadratic functions of time were not statistically significant for any of the groups. For the HD hypothesis, however, most variables were correlated with species richness of mosses and liverworts, being highly significant in the former group. Regarding the CLIMATE hypothesis, orographic mist layer (*MistL*) accounted for the highest proportion of data variability in both *S_M_* and *S_L_*. The negative effect of maximum temperature (*T_MAX_*) on liverwort species richness was also remarkable. Temperature seasonality (*T_S_*), although showing a lower correlation, was statistically significant for both mosses and liverworts.

**Table 1:** Univariate regressions explaining the variation in species richness of all Macaronesian bryophytes (*S_TOT_*), mosses (*S_M_*) and liverworts (*S_L_*) as a function of the predictors chosen for the Equilibrium Model of Island Biogeography (EMIB), the General Dynamic Model (GDM), the Habitat Diversity model (HD) and the Climatic Model (CLIMATE).

	All bryophytes (*S_TOT_*)		Mosses (*S_M_*)		Liverworts (*S_L_*)	
	*R^2^*	*F*	*R^2^*	*F*	*R^2^*	*F*
**EMIB**						
*A* (+)	0.132	2.59	0.222	4.85*	0.015	0.25
*D_M_*	0.002	0.43	0.009	0.16	0.102	1.94
*D_I_*	0.017	0.30	0.037	0.65	<0.001	0.00
*N*	0.079	1.46	0.064	1.16	0.087	1.62
**GDM**						
*T*	0.094	1.76	0.047	0.85	0.189	3.97†
*TT^2^*	0.191	1.88	0.154	1.46	0.243	2.57
**HD**						
*ELEV* (+)	0.405	11.58**	0.482	15.83***	0.201	4.26*
*sdELEV* (+)	0.505	17.32***	0.606	26.13***	0.242	5.42*
*SLOPEdiv*	0.045	0.79	0.050	0.89	0.023	0.39
*EZ* (+)	0.644	39.75***	0.579	23.39***	0.224	4.92*
**CLIMATE**						
*T_MAX_* (−)	0.195	4.12†	0.082	1.53	0.467	14.87**
*T_S_* (+)	0.275	6.45*	0.234	5.19*	0.294	7.10*
*P_MIN_*	0.004	0.07	0.005	0.09	0.103	1.96
*P_S_*	0.006	0.10	0.006	0.10	0.130	2.53
*P_ANN_* (+)	0.033	0.58	0.002	0.03	0.187	3.90†
*MistL* (+)	0.584	23.90***	0.503	17.20***	0.610	26.62***

† *p*<0.06,

* *p*<0.05,

** *p*<0.01,

*** *p*<0.001.

Variable codes: *A* (area), *D_M_* (distance to mainland), *D_I_* (distance to the nearest island), *N* (neighbour index); *T* (oldest geological age); *ELEV* (maximum elevation), *sdELEV* (standard deviation of elevation), *SLOPEdiv* (diversity of slopes); *EZ* (number of ecological zones); *T_MAX_* (maximum temperature of warmest month), *T_S_* (temperature seasonality), *P_MIN_* (precipitation of driest quarter), *P_S_* (precipitation seasonality), *P_ANN_* (annual precipitation), *MistL* (orographic mist layer).

The explanatory capacity of each variable (*R^2^*) and its statistical significance (*F*-test) are shown. The sign of the relationship is indicated under parenthesis after the predictor variable only when there is a significant effect on the dependent variable. The best fitting function (including significant quadratic functions) is shown in all cases, except for GDM for which both linear (*T*) and quadratic (*TT^2^*) functions of time are included as suggested in the literature [8], [10].

The subset of variables included in the best model for each hypothesis was also similar between mosses and liverworts (Table 2). The EMIB never exceeded 22% of explained variance, being weakly or even marginally significant, while GDM was statistically significant for both groups of bryophytes, particularly in mosses. Here note that while time alone is a poor predictor of species richness (Table 1), when included in a model with area (*A*) the two variables account for as much as 64% and 36% of data variation in moss and liverwort species richness, respectively (Table 2). HD seems to be particularly important for mosses, although CLIMATE was the model with the highest explanatory capacity for all groups (up to 77%). Given that the above mentioned differences between mosses and liverworts cannot be discerned when considering all species together (*S_TOT_*), henceforth we will focus on comparing the main findings for both taxonomic groups separately.

**Table 2:** Multiple regression results showing the best subset of predictors for each considered model (EMIB, GDM, HD and CLIMATE) to explain the between-island variation in species richness of Macaronesian bryophytes.

	*F*	*P*	*R^2^_adj_*	AIC*_C_*
**Total species richness (** ***S_TOT_*** **)**				
EMIB (*A*)	2.78	0.126	0.132	238.5
GDM (*A*, *TT* ^2^)	7.93	0.002	0.565	230.1
HD (*sdELEV*)	17.32	<0.001	0.565	227.8
CLIMATE (*T_MAX_*, *P_MIN_*, *MistL*)	15.67	<0.001	0.728	221.2
**Moss species richness (** ***S_M_*** **)**				
EMIB (*A*)	4.85	0.042	0.222	285.0
GDM (*A*, *TT* ^2^)	10.54	<0.001	0.638	212.0
HD (*sdELEV*)	26.13	<0.001	0.606	208.8
CLIMATE (*P_MIN_, MistL*)	17.07	<0.001	0.662	208.0
**Liverwort species richness (** ***S_L_*** **)**				
EMIB (*A*, *D_M_*)	2.85	0.088	0.219	198.8
GDM (*A*, *TT* ^2^)	3.83	0.032	0.363	197.5
HD (*sdELEV*)	5.42	0.033	0.242	196.1
CLIMATE (*T_MAX_, P_MIN_, MistL*)	19.28	<0.001	0.768	178.3

The best subset of variables that were chosen using the lowest sample size-corrected Akaike information criterion (AIC*_C_*) is shown in brackets. Adjusted *R^2^* values and its statistical significance according to the *F*-test are also shown. Model acronyms and variable codes as in Table 1.

**Figure 1:**
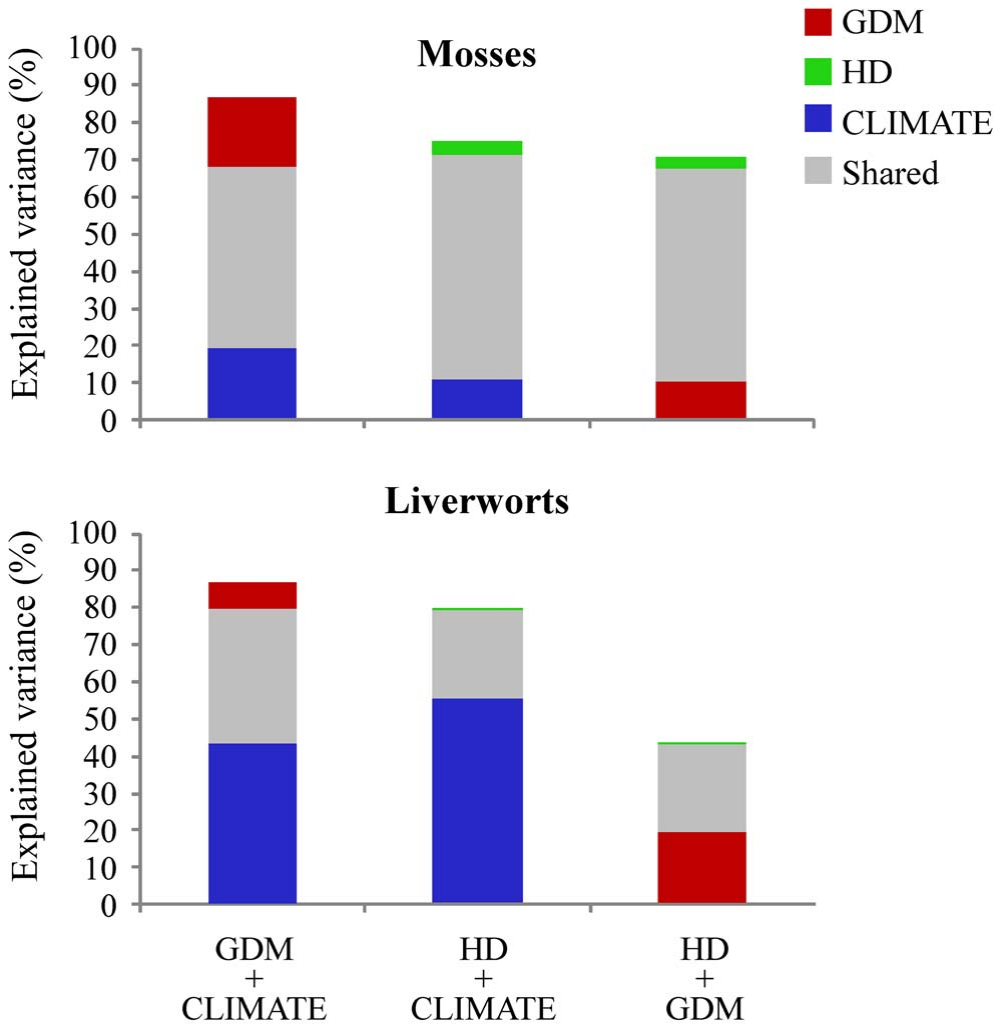
Partial regressions showing all pairwise comparisons between the models that better fit the species richness (Table 2). In all cases it is shown the percentage of variance explained exclusively by each model and the shared variance between each pair of models. Model acronyms as in Table 1.

Results from partial regressions including the hypotheses that seem to better explain species richness (see Table 2) indicated that the combined ‘GDM+CLIMATE’ model explained most of the variation in moss and liverwort richness (87.0% and 91.1%, respectively), followed by ‘HD+CLIMATE’ (71.5% and 79.8%) and ‘HD+GDM’ (71.1% and 43.6%) (Fig. 1). However, there were contrasting differences in the independent and shared effects of these hypotheses between both groups. In mosses, shared effects were very high in all pairwise model combinations (ranging between 48.9–60.6%), while pure effects were much lower (Fig. 1). Hence, when combined with the other hypotheses, CLIMATE alone explained around 11.0–19.2% of *S_M_*, GDM between 10.5–18.9% and HD no more than 3.5%. In the case of liverworts, however, around half of the variability in species richness was accounted exclusively by CLIMATE, while the independent effects of both GDM and HD were weaker (up to 19% and 0.4% of explained variance, respectively). Note that pairwise comparisons between GDM and HD hypotheses showed a relatively higher contribution of the former over the latter in both taxonomic groups.

The spatial analyses evidenced that the above models account for almost all spatially-structured variation in the data. Model residuals were not significantly correlated with latitude in any case except for the HD model in liverworts (Spearman *r* = 0.57, *p* = 0.01). In fact, there were only three statistically significant autocorrelation values in model residuals (in the case of GDM for mosses and of GDM and CLIMATE for liverworts; see Tables S3.1, S3.2 in File S3). Further, archipelago idiosyncrasies seem not to have affected model estimates, since SAR models were consistent with OLS regressions (File S3) and the statistical significance of the parameters for the variables included in OLS regressions remained similar. Once the spatial structure was taken into account, the predictive ability of SAR models (*R^2^*) increased only slightly (compare Table S3.3 in File S3 with Table 2).

## Discussion

Although interpreting patterns of species diversity is a recurrent issue in island biogeography, the number of studies examining different factors altogether is surprisingly low (e.g. [11], [18], [42], [43]). For instance, Kreft et al. [19] related worldwide patterns of vascular plant island species richness to geographic, topographical and climatic characteristics, filtering also by the geological origin of islands. However, these authors did not account for the different intra-archipelago relationships expected when crossing biogeographical regions [20] and their meta-analyses did not disentangle the combined and independent effect of each factor. Our findings suggest that climate and habitat are the most relevant factors in explaining bryophyte richness differences on oceanic islands. These suggest that the factors operating during species establishment may be relatively more important than dispersal during the colonization process, at least in the case of the Macaronesian region.

### The role of climate and habitat in bryophyte diversity

Our results show that the variation of bryophyte species richness between the Macaronesian islands can be relatively well predicted by climatic conditions, particularly with those favouring higher humidity. In this respect, it is well known that water availability is an important component of several key ecophysiological processes in bryophytes [25]. One could assume that water availability is of major importance during the colonization of oceanic islands by bryophytes. Since long-distance dispersal (LDD) is mostly driven by spores (see below), water becomes essential since both sexual reproduction and spore germination depends on it. Once the populations are effectively established on an island, the availability of water at adequate growing temperatures becomes crucial to achieve a positive net photosynthetic rate over time, otherwise the plants enter dormancy upon drying (reviewed by [44]). Consequently, the time of survival when an individual colony or shoot remains dry is also dependent on the periods with mild temperatures. It follows that the role of water availability (and secondarily temperature) to maintain populations and ultimately avoid local species extinctions could be the main reason to explain the relevance of the considered climatic variables in our study.

Contrary to most vascular plants, bryophytes regulate water uptake mainly by capillarity and since they are usually small, their survival may be more restricted by the frequency rather than by the volume of rainfall [45]. Actually, dew or cloud water deposition is often sufficient to remoisten most bryophyte species. In agreement with this idea, our results show that orographic mist layer (*MistL*), together with lower values of minimum precipitation (*Pmin*) and maximum temperature (*Tmax*) correlate with higher values of species richness. Such climate is typical of the Azorean archipelago and Madeira island but also, to a lesser extent, of La Gomera and La Palma in the Canaries [28]. The importance of air humidity is clearly evident in (sub-) tropical rainforests [46], especially in canopy epiphytes (see [47]), whose species diversity may indeed be comparable to some Azorean islands where more than 25 species can occur in plots of only 30 cm×30 cm [38].

However, the impact of climate must be broadly analyzed, taking into account the landscape conditions where the species grow [48]. Our results show high correlations of habitat diversity with bryophyte diversity, providing an indirect signal for a certain degree of habitat specialization in Macaronesian bryophytes, in agreement with evidences that closely related species from this group may coexist sympatrically in separate niche spaces [49]. This implies that the high dispersal ability of bryophytes does not lead necessarily to habitat generalism, as for other taxa with strong vagility (e.g. [12]). Strikingly, habitat diversity seems to be as important as climate in our case, particularly for the Macaronesian moss flora. However in this study, most of the variables representing habitat diversity are eminently topographical and hence somehow correlated with island ontogeny (i.e. time) as well as with climatic predictors resulting in strong shared effects and making it difficult to disentangle their specific influence on species richness (see Fig. 1). In fact, the explanatory power of this factor remains similar even when we consider the number of ecological zones (*EZ*) as a surrogate of habitat diversity, probably because the major vegetation formations in Macaronesia are strongly structured in altitudinal belts (see File S1); the number of ecological zones is highly correlated with both *sdELEV* (*r* = 0.94) and *ELEV* (*r* = 0.92), thus being a surrogate for mesoscale climate gradients as well as for habitat diversity *per se* (see [50]).

The contrasting patterns found in liverworts and mosses could at first be related with their distinct ability to produce sexual and asexual diaspores, but no differences in the expression of several life-history traits between the two groups were detected in a suite of oceanic archipelagos [51]. The apparently higher climatic sensitivity of liverworts compared to mosses could then be understood by their lower desiccation tolerance [52], especially notable in leafy liverworts due to their life-form traits [53]. This is mirrored by the high sensitivity of the group to human-induced disturbances [39]. Perhaps due to this, mosses can be found in a comparatively wider range of landscapes, including grasslands and other man-made habitats [25], while liverworts seem to be more dependent on sheltered habitats like forests as compared with other open landscapes. In fact, liverwort richness at the Azorean native forests is higher than that of mosses above 600 m a.s.l. [38]. Further studies are necessary to confirm such higher habitat specificity across the latitudinal gradient provided by the Macaronesian archipelagos.

### Effects of island isolation, area and time

Long-distance dispersal (LDD) is a rare and stochastic event [54], although its prevalence over long time periods may be common [4]. In the case of bryophytes, LDD may occur only occasionally because spore production seems to be highly constrained due to unsuccessful sexual reproduction and even when occurring, spore release typically falls within the first tens of meters [55] (but see [56]). Hence, asexual propagation is often the most frequent way of dispersion [35]. This argument is also supported by an increasing number of molecular evidences showing that population connectivity at local or even landscape scales is hindered by dispersal limitation (e.g. [57], [58]). In line with this rationale, significant shifts in life-history traits towards decreased sporophyte production and increased production of specialized asexual diaspores on oceanic islands recently pointed to a global loss of LDD ability in oceanic bryophyte floras [51]. It must be acknowledged, however, that spore production may be overlooked when fieldwork has not been sufficiently intensive (e.g. [59]). The ability of taxa to undergo LDD via asexual propagules (usually larger than 50 µm) is still not clearly understood and, contrary to classical studies with spores [60], [61], little is known about the maximum distances that vegetative propagules might actually travel (cf. [62]). Simulation studies have recently pointed out that above a diameter of 20 µm wind dispersal of microbes between continents becomes increasingly unlikely, and it does not occur at all for those of 60 µm diameter [63]. Hence, once airborne, bryophyte spores can be virtually transported large distances by wind, so other establishment impediments such as edge colonization or gene surfing [64] and niche specialization [65] could be as limiting factors as dispersal *per se*.

Here we are assuming that LDD is likely achieved by spores because they are resilient, microscopic and released in several millions, with some of them eventually being able to colonize an island in the very long range. This mechanism might particularly apply to archipelagos such as the Macaronesian islands [36], which are relatively less isolated and exhibited a higher connectivity with the continental sources in the past, due to the presence of a higher number of emerged islands that remain today as submersed seamounts [66]. Our results are in line with these arguments showing that the limited contribution of the Equilibrium Model of Island Biogeography in Macaronesian bryophytes can be justified at least by the negligible effect of geographic isolation we expected. Different studies have shown similar results in bryophytes [17], [67], [68], as well as in other organisms that disperse passively by spores, for which wind connectivity seems to be more important than geographic proximity between land masses [69].

By contrast, the effect of area on bryophyte species richness has been either supported or not in different studies, both in the case of islands and isolated patches on fragmented landscapes (e.g. [22], [37], [67], [70]). Our results show that the General Dynamic Model –which is ultimately an extension of that originally proposed by the EMIB– still exerts significant predictive power over island richness after accounting for other factors, particularly in the case of mosses (cf. [17]). The variation of island area through time is not expected to affect the speciation process in this taxon because of its high dispersal potential, but both immigration and extinction are probably influenced by the changes in habitat diversity through the island's ontogeny, as predicted by this theory [8]. The few studies that have evaluated the effects of island age on whole floras of spore-dispersed plants show a comparatively lower predictive power of this variable than area and habitat diversity [17], [29], [67]. At this point, however, one could argue that it is very difficult to disentangle the effect of HD and GDM hypotheses because time is implicitly accounting for the changes in island topography which, in turn, is also correlated with area. In fact, we observe strong shared effects between both hypotheses although, as we mentioned above, this could be related with the variables chosen to represent habitat diversity. Hence, what we may interpret from our results is that neither time nor area (nor topography) alone explain as much variability in the data as both factors together (around 40% or even 60% in liverworts and mosses, respectively).

In spite of its importance, the precise relationship between area and species richness is still under debate. Several alternative hypotheses have been proposed to explain the importance of this relationship on extinction, colonization and speciation rates or even stochastic processes [71], among which its correlation with habitat diversity seems to apply in different taxa (see [9] and references therein). Yet, separating the effects of these factors is statistically challenging, and comparatively fewer studies have analyzed the influence of the ‘effective area’ over species richness, that is, the relevance of area of suitable habitats [18], [42].

### Some notes of caution

Determining which predictors are the most ecologically meaningful for a particular taxon is always difficult to scrutinize by the available statistical techniques, particularly because the obtained relationships depend basically on (i) the geographic extent of the study region, (ii) the dataset (i.e. sample size) and (iii) the existence of multicollinearity among predictors. These problems are exacerbated in the case of island biogeography due to the combination of small sample sizes and different relationships between species richness and island characteristics among different archipelagos [21]. However, our objective here was to choose the best combination of variables to represent each hypothesis – i.e. to account for data variation in species richness – rather than selecting the individual predictors that are most biologically important. In spite of this, we mentioned this issue and, by comparing also with analyses including the Cape Verde archipelago (File S1), we could say that at least orographic mist layer together with temperature seem to be important for these organisms (see Tables 1 and 2, and Table S1.1 and S1.2 in File S1). Few studies have proved experimentally the influence of mist layer because quantitative data on air humidity are often hard to obtain (e.g. [46], [72]), hence using typically indirect measures like bryophyte cover itself [47]. Further investigation is however required to confirm the role of mist precipitation by incorporating an actual proxy (e.g. frequency or volume of mist precipitation), testing its independent and combined effect with annual precipitation.

Obviously, widening the spatial extent of the study entails an overall higher contribution of climate – in both groups of bryophytes – due to a stronger latitudinal gradient (Table S1.1, S1.2 and Fig. S1.1 in File S1). Nevertheless, habitat variables have a notably contribution explaining the richness of mosses (Table S1.1, S1.2 in File S1), while showing a high shared variance with climate (Fig. S1 in File S1). Nonetheless, the consistency between the results obtained with ordinary least squares models and spatial autoregressive regressions indicate that differences in the spatial positioning of islands are not conditioning the main patterns found at the Macaronesian extent.

## Conclusions

Our findings indicate that large-scale variations in the Macaronesian bryophyte diversity are highly influenced by environmental factors but also, at least in the case of mosses, by factors related with the island ontogeny. This could be the case for other taxa with high dispersal ability. We have also shown strongly different macroecological patterns between mosses and liverworts, reinforcing the idea that not only dispersal ability, but also different ecophysiological responses of these two evolutionarily distinct lineages, are probably shaping the distribution of species diversity. Our results evidence the seeming importance of climate, in particular orographic mist layer, for liverwort diversity, while in mosses this factor has a similar or, at least, indiscernible effect to that of habitat or even the geologic ontogeny. These results point to a presumably large relevance of the establishment process on the island diversity of spore-dispersed plants. Future studies, using broader spatial extents are required to generalize these conclusions.

## Methods

### Area of study

The five Macaronesian archipelagos (Azores, Madeira, Selvagens, Canaries and Cape Verde) lie in the North Atlantic Ocean, covering a maximum latitudinal extension of almost 3000 km. Although all these archipelagos have a volcanic origin, the geographical characteristics of the islands vary widely in both size (ranging from 3 km^2^ to more than 2000 km^2^) and isolation (oscillating from less than 100 km to about 1800 km in distance to the nearest continent). Their maximum geological age also differs significantly among islands, from the youngest island of Pico in the Azores (less than half a million years) to the Selvagem Grande in the Selvagens archipelago that dates back to the Miocene (27 Ma). It is known, however, that a much older and interconnected “Palaeo-Macaronesia” existed during the Paleocene (60 Ma), most of which remains today as submersed seamounts [66]. There are also evident climatic differences between archipelagos along the large latitudinal gradient they form, from the temperate oceanic conditions of the Azores to the Mediterranean climate of Madeira, Selvagens and Canary Islands. The most extreme conditions for bryophyte survival occur in Cape Verde, where tropical arid climate prevails, and in the Canarian islands of Lanzarote and Fuerteventura, all showing desert affinities due to the Sub-Saharan influence. Despite such contrasting island features, there are some biotic elements shared between most archipelagos among which the evergreen laurel forests (or laurisilva) are probably the best representative example for Azores, Madeira and the Canaries. Although most of these forest areas were highly reduced after the Pleistocene glaciations and current human activity, they present the optimal habitat conditions for attaining maximum levels of bryophyte diversity (e.g. [73]).

### Data compilation

We calculated total species richness (*S_TOT_*) per island using recent checklists updated with some relevant references (see further details in [22]). As the species checklist from Selvagens and some Cape Verde islands cannot be considered reliable [22], we focus our analyses on the main islands (*n* = 19) of Azores, Madeira and the Canaries (but see also File S1, where we show additional analyses including some Cape Verde islands that could be comparable in terms of inventories). We also run separate analyses using the species richness of the two dominant groups of mosses (*S_M_*) and liverworts (*S_L_*) because they normally present different ecophysiological responses [40]. In total, our database included all the 729 bryophyte species recorded in the four Macaronesian archipelagos. Out of these, 505 are mosses, 218 liverworts and 6 hornworts.

Fifteen predictor variables representing geography, time, habitat diversity and climate were used to evaluate the relevance of the four hypotheses formulated above (see File S2 for further details on computation and data sources). For H_1_ (i.e. EMIB), we compiled data on island area (*A*), distance to mainland (*D_M_*) and distance to the closest island (*D_I_*). We also calculated the neighbour index (*N*) for all the islands as proposed by Kalmar and Currie [11], to account for the combined effects of the area and distance of nearby islands. For H_2_ (i.e. GDM), apart from area we obtained the time elapsed since island formation (*T*) for each island. For H_3_ (i.e. HD), we used the number of main ecological zones in the islands (*EZ*) as well as three topographical surrogates: maximum elevation (*ELEV*), standard deviation of elevation (*sdELEV*) and diversity of slopes (*SLOPEdiv*). Finally, for H_4_ (i.e. CLIMATE), we used six variables accounting for extreme, average and intra-annual variation of precipitation and temperature that are of particular importance for bryophyte distribution: maximum temperature of warmest month (*T_MAX_*), precipitation of driest quarter (*P_MIN_*), temperature seasonality (*T_S_*), precipitation seasonality (*P_S_*), annual precipitation (*P_ANN_*) and an index of horizontal precipitation as surrogate of orographic mist layer (*MistL*).

### Statistical analyses

We used ordinary least squares (OLS) regressions to evaluate the influence of the different predictors on bryophyte species richness. Prior to the analyses, all these variables were standardized to zero mean and one standard deviation to remove the effect of different measurement scales. We first explored the univariate relationships between the considered dependent variables (*S_TOT_*, *S_M_* and *S_L_*) and each one of the 15 predictors. We evaluated both linear and curvilinear (quadratic) relationships selecting for subsequent analyses the function that maximized the explained variance in the response variable. In the case of GDM we included both the linear (*T*) and quadratic (*TT^2^*) functions of time as suggested in the literature [8], [10]. We then selected the best subset of predictors representing each hypothesis through multiple regressions, using the Akaike information criterion corrected for small sample sizes (AIC*_C_*) to compare alternative models. Lower AIC*_C_* values indicate a compromise between higher model fit and lower complexity, so for each hypothesis we chose as best model the one with the minimum AIC*_C_* [74]. In the case of GDM, we applied directly the *ATT* ^2^ model proposed by Whittaker et al. (2008) that assumes an unimodal response of species richness to time, also accounting for the positive and monotonic influence of area. We additionally reported conventional statistics (*R^2^* and *F*-test) for all the obtained models.

To ascertain whether the combined effects of the different models selected previously contribute to increase significantly the explained variance, we made pairwise comparisons between the best models chosen for each hypothesis. To separate the single and combined effects of the different models, we used partial regression analyses [75]. Unlike standard regression methods, this technique allows disentangling the proportion of explained variance that can be attributed exclusively to one set of factors once the effect of other sets has been controlled for, assuming that combined effects reflect the shared variance that cannot be unequivocally attributed to any of the individual sets of predictors.

In order to examine whether archipelago' idiosyncrasies non-strictly related with the abiotic island characteristics here considered may be disrupting our interpretation of species richness patterns, we followed three alternative but complementary approaches. As the low number of observations hinders the use of a qualitative variable reflecting the different archipelagos as a fixed factor, we took advantage of the strong latitudinal gradient in the location of the different Macaronesian archipelagos to examine if a spatial pattern remains in our models. To do this we correlated model residuals with latitude, also evaluating the statistical significance of Moran's *I* autocorrelation values using Monte Carlo methods (see [76]). We also run simultaneous autoregressive regressions (SAR) taking explicitly into account the spatial coordinates in the analysis as a connectivity matrix [77].

All analyses were performed in SAM v4.0 [78] (available at www.ecoevol.ufg.br/sam/).

## Supporting Information

File S1 Additional analyses incorporating the Cape Verde archipelago.
(DOCX)

File S2 Abiotic characteristics of the Macaronesian islands considered, and species richness of all bryophytes (*S_TOT_*), mosses (*S_M_*) and liverworts (*S_L_*).
(DOCX)

File S3 Spatial autocorrelation of model residuals and spatial autoregressive regressions (SAR) alternative to typical ordinary least squares (OLS) regression models.
(DOCX)
