# Supplementary material for: Geographical, Temporal and Environmental Determinants of Bryophyte Species Richness in the Macaronesian Islands
Source: PLoS One. 2014 Jul 8;9(7):e101786. doi: 10.1371/journal.pone.0101786 (PMC4086965; doi:10.1371/journal.pone.0101786)
Supplement: File S1 — Additional analyses incorporating the Cape Verde archipelago. (DOCX) [file pone.0101786.s001.docx]

**Supporting Information S1.** Additional analyses incorporating the Cape Verde archipelago.

After evaluating the reliability of all island inventories in the Macaronesian region (following [1]), four out of eight Cape Verde islands with available data could be considered comparable to the rest of islands in terms of species richness (Supporting Information S2; see further details in [2]). We hence repeated the same analyses as in the main text of the manuscript including also these islands (*n* = 24).

When including Cape Verde, all results showed a relative higher contribution of climatic variables to account for variation in species richness data (Tables S1.1, S1.2 and Fig. S1). In particular, orographic mist layer (*MistL*) was the climatic variable that showed the highest correlation in univariate analyses (Table S1.1) and it was always selected among the best subset of variables to represent the CLIMATE hypothesis (Table S1.2). These findings, together with those resulting from analyses in the main text, may suggest that orographic mist layer is one of the most important predictors of bryophyte species richness in the Macaronesian region. Habitat diversity (HD), however, took an important role when including the four Cape Verde islands with reliable inventories, although its influence on bryophyte diversity seems to be still higher in mosses than liverworts (Fig. S1). In particular, HD showed a much higher contribution when compared with the GDM (cf. Fig. 1 in the main text). Also, note here that the number of ecological zones (*EZ*) was much more explicative of richness patterns than the other topographic surrogates. The contribution of the General Dynamic Model of oceanic island biogeography (GDM) was only marginally significant (Table S1.2) and had a low relative weight when compared with the other hypotheses (Fig. S1).

Compared with analyses in the main text, the seeming higher importance of climatic variables found at this spatial extent is likely due to the greater latitudinal gradient covered when including the most southern Cape Verde archipelago. Despite there are similar levels of data variation between islands attributes (see Supporting Information S2), between-archipelago climatic differences are stronger than those occurring in the other hypotheses. Still, HD showed a strong shared variance with CLIMATE but a higher predictive ability than GDM. The corollary is that assessing the relative importance of geographic, geologic and environmental factors depends on the geographic extent covering the study region. This makes difficult establishing generalization about biogeographic processes, even for organisms with a priori no dispersal constraints but with strong environmental dependence instead.

**Table S1.1** Univariate regressions explaining the variation in species richness of all Macaronesian bryophytes (*S_TOT_*), mosses (*S_M_*) and liverworts (*S_L_*) as a function of the predictors chosen for: (i) the Equilibrium Model of Island Biogeography (EMIB), (ii) the General Dynamic Model (GDM), (iii) the Habitat Diversity model (HD) and (iv) the climatic model (CLIMATE). The explanatory capacity of each variable (*R^2^*) and its statistical significance (*F*-test) are shown. The sign of the relationship is indicated under parenthesis after the predictor variable only when there is a significant effect on the dependent variable. The best fitting function (including significant quadratic functions) is shown in all cases, except for GDM for which both linear (*T*) and quadratic (*TT^2^*) functions of time are included as suggested in the literature [3], [4].

|  | **All bryophytes (*S_TOT_*)** | | **Mosses (*S_M_*)** | | **Liverworts (*S_L_*)** | |
| --- | --- | --- | --- | --- | --- | --- |
|  | *R^2^* | *F* | *R^2^* | *F* | *R^2^* | *F* |
| **EMIB** |  |  |  |  |  |  |
| *A* | 0.077 | 1.83 | 0.139 | 3.55 | 0.008 | 0.18 |
| *D_M_* | 0.017 | 0.38 | <0.001 | 0.00 | 0.112 | 2.77 |
| *D_I_* | 0.089 | 2.14 | 0.109 | 2.68 | 0.046 | 1.05 |
| *N* | 0.027 | 0.60 | 0.024 | 0.55 | 0.026 | 0.58 |
| **GDM** |  |  |  |  |  |  |
| *T* | 0.047 | 1.08 | 0.023 | 0.52 | 0.097 | 2.37 |
| *TT^2^* | 0.055 | 0.56 | 0.023 | 0.25 | 0.116 | 1.38 |
| **HD** |  |  |  |  |  |  |
| *ELEV* (+) | 0.141 | 3.61 | 0.197 | 5.40^*^ | 0.050 | 1.16 |
| *sdELEV* (+) | 0.163 | 4.28^*^ | 0.231 | 6.62^*^ | 0.056 | 1.29 |
| *SLOPEdiv* | 0.013 | 0.30 | 0.020 | 0.46 | 0.003 | 0.06 |
| *EZ* (+) | 0.644 | 39.75^***^ | 0.699 | 51.00^***^ | 0.460 | 18.76^***^ |
| **CLIMATE** |  |  |  |  |  |  |
| *T_MAX_* (–) | 0.287 | 8.87^**^ | 0.196 | 5.37^*^ | 0.435 | 16.94^***^ |
| *T_S_* (+) | 0.531 | 24.89^***^ | 0.464 | 19.03^***^ | 0.569 | 29.09^***^ |
| *P_MIN_* (+) | 0.094 | 2.29 | 0.036 | 0.81 | 0.246 | 7.19^*^ |
| *P_S_* (–) | 0.334 | 11.03^**^ | 0.232 | 6.66^*^ | 0.493 | 21.41^***^ |
| *P_ANN_* (+) | 0.165 | 4.34^*^ | 0.083 | 1.98 | 0.343 | 11.51^**^ |
| *MistL* (+) | 0.524 | 24.17^***^ | 0.484 | 20.67^***^ | 0.515 | 23.39^***^ |

^†^ *p*<0.06, * *p*<0.05, ** *p*<0.01, *** *p*<0.001.

Variable codes: *A* (area), *D_M_* (distance to mainland), *D_I_* (distance to the nearest island), *N* (neighbour index); *T* (oldest geological age); *ELEV* (maximum elevation), *sdELEV* (standard deviation of elevation), *SLOPEdiv* (diversity of slopes); *EZ* (number of ecological zones); *T_MAX_* (maximum temperature of warmest month), *T_S_* (temperature seasonality), *P_MIN_* (precipitation of driest quarter), *P_S_* (precipitation seasonality), *P_ANN_* (annual precipitation), *MistL* (orographic mist layer).

**Table S1.2** Multiple regression results showing the best subset of predictors for each considered hypothesis (EMIB, GDM, HD and CLIMATE) to explain the between-island variation in species richness of Macaronesian bryophytes. The best subset of variables that were chosen using the lowest sample size-corrected Akaike information criterion (AIC*_C_*) is shown in brackets. Adjusted *R^2^* values and its statistical significance according to the *F*-test are also shown. Model acronyms and variable codes as in Table S1.1.

|  | *F* | *P* | *R^2^_adj_* | AIC*_C_* |
| --- | --- | --- | --- | --- |
| **Total species richness (*S_TOT_*)** |  |  |  |  |
| EMIB (*A*, *D_M_*) | 2.80 | 0.083 | 0.175 | 306.0 |
| GDM (*A*, *TT^2^*) | 2.20 | 0.120 | 0.176 | 308.1 |
| HD (*EZ*) | 39.75 | <0.001 | 0.644 | 284.0 |
| CLIMATE (*P_MIN_*, *P_S_*, *MistL*) | 21.94 | <0.001 | 0.745 | 280.0 |
| **Moss species richness (*S_M_*)** |  |  |  |  |
| EMIB (*A*) | 3.55 | 0.073 | 0.139 | 284.9 |
| GDM (*A*, *TT^2^*) | 2.79 | 0.067 | 0.228 | 286.2 |
| HD (*EZ*) | 23.39 | <0.001 | 0.579 | 259.7 |
| CLIMATE (*P_MIN_*, *T_S_*, *MistL*) | 21.64 | <0.001 | 0.742 | 259.9 |
| **Liverwort species richness (*S_L_*)** |  |  |  |  |
| EMIB (*A*, *D_M_*) | 3.31 | 0.056 | 0.205 | 256.1 |
| GDM (*A*, *TT^2^*) | 1.59 | 0.224 | 0.115 | 260.8 |
| HD (*EZ*) | 4.92 | 0.040 | 0.224 | 245.0 |
| CLIMATE (*P_MIN_, P_S_, MistL*) | 21.17 | <0.001 | 0.738 | 231.6 |

**Figure S1** Partial regressions results showing the explained variance of bryophyte species richness when considering CLIMATE together with each of the other three models (EMIB, HD and GDM). In all cases it is shown the percentage of variance explained exclusively by each model and the shared variance between each pair of models. Model acronyms as in Table S1.1.

**
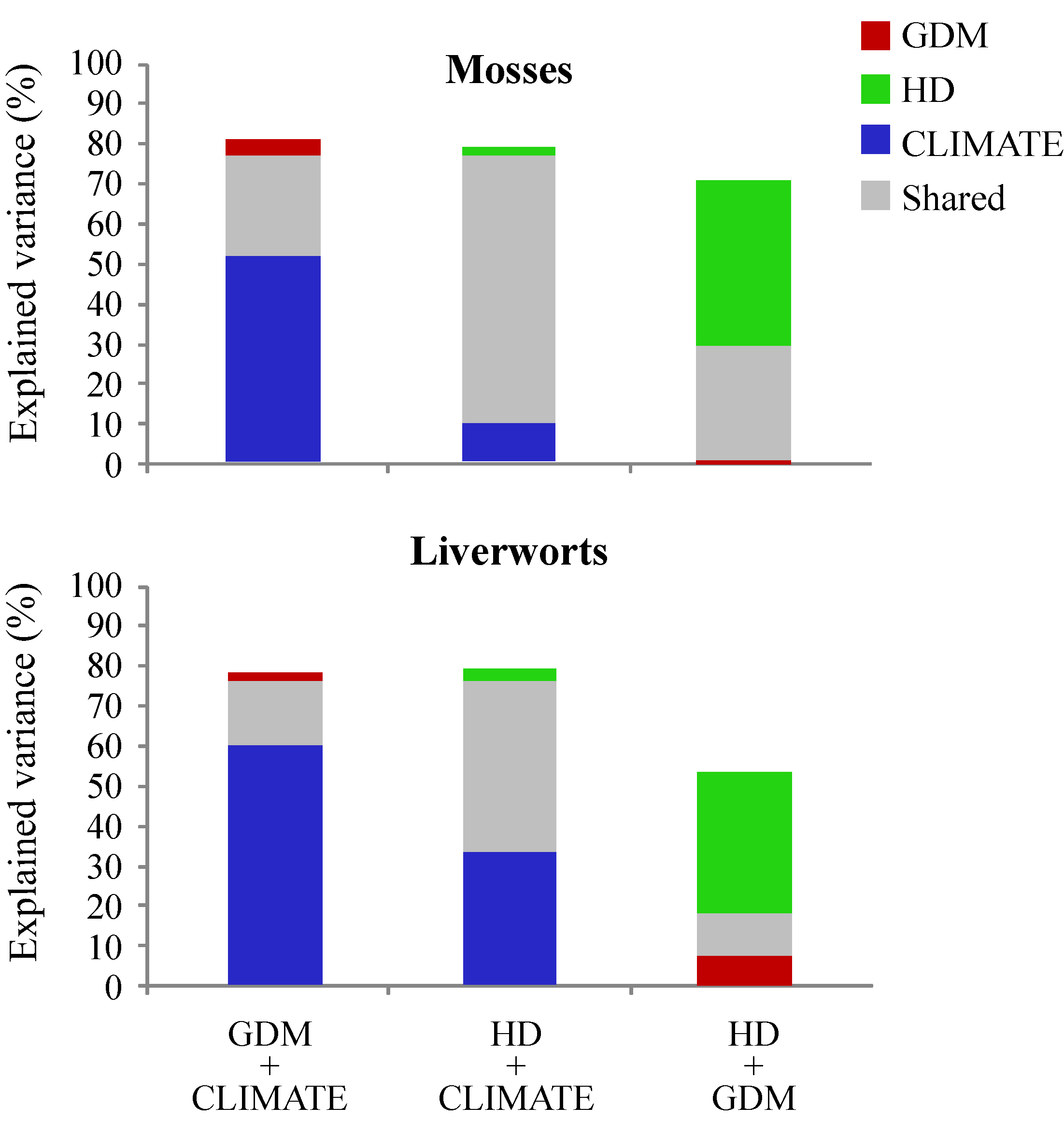
**

**REFERENCES**

1. Santos AMC, Jones OR, Quicke DLJ, Hortal J (2010) Assessing the reliability of biodiversity databases: identifying evenly inventoried island parasitoid faunas (Hymenoptera: Ichneumonoidea) worldwide. Insect Conservation and Diversity 3: 72-82.

2. Aranda SC, Gabriel R, Borges PAV, Santos AMC, Hortal J, et al. (2013) How do different dispersal modes shape the species-area relationship? Evidence for between-group coherence in the Macaronesian flora. Global Ecol Biogeogr 22: 483-493.

3. Whittaker RJ, Triantis KA, Ladle RJ (2008) A general dynamic theory of oceanic island biogeography. J Biogeogr 35: 977-994.

4. Whittaker RJ, Triantis KA, Ladle RJ (2010) A general dynamic theory of oceanic island biogeography: extending the MacArthur–Wilson theory to accommodate the rise and fall of volcanic islands. In: Losos JB, Ricklefs RE, editors. The theory of island biogeography revisited Princeton, NJ: Princeton University Press. pp. 88-115.
