# Supplementary material for: Geographical, Temporal and Environmental Determinants of Bryophyte Species Richness in the Macaronesian Islands
Source: PLoS One. 2014 Jul 8;9(7):e101786. doi: 10.1371/journal.pone.0101786 (PMC4086965; doi:10.1371/journal.pone.0101786)
Supplement: File S2 — Abiotic characteristics of the Macaronesian islands considered, and species richness of all bryophytes (STOT), mosses (SM) and liverworts (SL). (DOCX) [file pone.0101786.s002.docx]

**Supporting Information S2.** Abiotic characteristics of the Macaronesian islands considered, and species richness of all bryophytes (*S_TOT_*), mosses (*S_M_*) and liverworts (*S_L_*). Information is organized according to each hypothesis: the Equilibrium Model of Island Biogeography (EMIB), the General Dynamic Model (GDM), the Climatic Model (CLIMATE) and the Habitat Diversity model (HD).

|  |  |  |  | EMIB | | | |  | GDM |  | CLIMATE | | | | | |  | HD | | |  | |  |
| --- | --- | --- | --- | --- | --- | --- | --- | --- | --- | --- | --- | --- | --- | --- | --- | --- | --- | --- | --- | --- | --- | --- | --- |
| Island | *S_TOT_* | *S_M_* | *S_L_* | *A*  *(*km^2^) | *D_M_*  (km) | *D_I_*  (km) | *N* |  | *T*  (Ma) |  | *T_MAX_*  (ºC) | *P_MIN_*  (mm) | *T_S_*  (%) | *P_S_*  (%) | *P_ANN_*  (mm) | *MistL* |  | *ELEV*  (m) | *sdELEV*  (m) | *SLOPE*  *div* | | *EZ* | |
| COR | 178 | 109 | 66 | 17 | 1858 | 18 | 0.42 |  | 0.73 |  | 24.9 | 174.3 | 30.0 | 37.1 | 1338.5 | 62.00 |  | 718 | 171.79 | 2.33 | | 2 | |
| FLO | 272 | 162 | 106 | 143 | 1864 | 18 | 0.07 |  | 2.16 |  | 24.1 | 191.2 | 30.7 | 36.9 | 1492.9 | 65.40 |  | 913 | 210.25 | 2.02 | | 4 | |
| FAI | 281 | 169 | 107 | 173 | 1657 | 6 | 9.2 |  | 0.73 |  | 24.2 | 147.9 | 29.9 | 33.5 | 1076.5 | 46.60 |  | 1043 | 228.88 | 1.55 | | 5 | |
| PIC | 279 | 156 | 119 | 436 | 1610 | 6 | 4.34 |  | 0.25 |  | 23.5 | 149.1 | 30.2 | 34.7 | 1123.2 | 59.00 |  | 2351 | 363.39 | 1.64 | | 6 | |
| GRA | 129 | 81 | 46 | 62 | 1596 | 36 | 0.46 |  | 2.50 |  | 24.6 | 134.1 | 29.0 | 38.3 | 1093.8 | 5.70 |  | 398 | 89.97 | 1.51 | | 2 | |
| SJO | 308 | 176 | 128 | 246 | 1584 | 18 | 1.72 |  | 0.55 |  | 23.9 | 139.8 | 29.7 | 37.0 | 1111.3 | 70.00 |  | 1067 | 219.80 | 2.01 | | 4 | |
| TER | 355 | 208 | 142 | 400 | 1520 | 37 | 0.34 |  | 3.52 |  | 23.8 | 128.1 | 29.1 | 43.6 | 1166.5 | 45.20 |  | 1020 | 206.42 | 1.33 | | 4 | |
| SMI | 355 | 227 | 124 | 750 | 1368 | 80 | 0.06 |  | 4.01 |  | 24.2 | 115.6 | 28.6 | 39.3 | 1044.4 | 48.80 |  | 1103 | 208.01 | 1.75 | | 5 | |
| SMA | 211 | 136 | 72 | 97 | 1376 | 80 | 0.14 |  | 8.12 |  | 25.2 | 79.1 | 28.1 | 45.5 | 765.4 | 14.70 |  | 587 | 103.38 | 1.75 | | 4 | |
| FUE | 130 | 90 | 40 | 1725 | 95 | 11 | 5.73 |  | 20.00 |  | 26.9 | 0.0 | 24.9 | 90.8 | 120.5 | 0.33 |  | 807 | 120.58 | 1.37 | | 4 | |
| CAN | 306 | 224 | 77 | 1532 | 195 | 61 | 0.80 |  | 14.50 |  | 26.6 | 3.6 | 26.6 | 84.9 | 250.6 | 38.33 |  | 1950 | 411.40 | 2.04 | | 6 | |
| GOM | 291 | 193 | 94 | 378 | 333 | 28 | 2.86 |  | 12.00 |  | 26.5 | 4.6 | 28.7 | 85.2 | 373.4 | 40.34 |  | 1484 | 333.56 | 2.17 | | 7 | |
| HIE | 195 | 135 | 57 | 278 | 381 | 61 | 0.47 |  | 1.10 |  | 26.3 | 2.7 | 28.0 | 87.8 | 311.2 | 44.79 |  | 2423 | 389.49 | 2.04 | | 7 | |
| LAN | 114 | 88 | 25 | 796 | 125 | 11 | 11.98 |  | 16.00 |  | 27.0 | 0.3 | 26.1 | 89.2 | 148.4 | 0.15 |  | 670 | 125.90 | 1.06 | | 2 | |
| PAL | 348 | 247 | 95 | 729 | 414 | 57 | 0.50 |  | 1.70 |  | 25.2 | 5.6 | 30.7 | 84.7 | 441.0 | 60.13 |  | 2425 | 536.37 | 2.11 | | 8 | |
| TEN | 430 | 296 | 128 | 2058 | 286 | 28 | 1.03 |  | 8.00 |  | 25.5 | 7.1 | 31.5 | 83.6 | 436.6 | 53.84 |  | 3718 | 708.35 | 1.83 | | 9 | |
| DES | 90 | 56 | 30 | 15 | 599 | 24 | 1.24 |  | 5.01 |  | 24.8 | 15.4 | 23.6 | 71.7 | 531.4 | 0.00 |  | 442 | 121.32 | 2.41 | | 2 | |
| MAD | 510 | 333 | 171 | 740 | 639 | 24 | 0.08 |  | 4.63 |  | 22.8 | 28.7 | 26.8 | 67.0 | 682.1 | 64.91 |  | 1850 | 403.29 | 2.26 | | 6 | |
| PSA | 113 | 45 | 64 | 40 | 633 | 39 | 0.03 |  | 14.00 |  | 24.7 | 17.4 | 24.9 | 66.4 | 431.2 | 0.00 |  | 517 | 82.66 | 1.85 | | 2 | |
| NIC | 72 | 58 | 14 | 343 | 721 | 17 | 0.40 |  | 6.20 |  | 27.1 | 0.0 | 16.6 | 144.8 | 219.4 | 5.17 |  | 1304 | 209.79 | 2.00 | | 2 | |
| FOG | 54 | 36 | 17 | 476 | 725 | 18 | 0.56 |  | 5.00 |  | 24.5 | 0.7 | 14.4 | 150.5 | 388.8 | 49.49 |  | 2829 | 630.42 | 1.86 | | 2 | |
| ANT | 109 | 91 | 17 | 779 | 834 | 13 | 1.28 |  | 7.50 |  | 24.4 | 0.5 | 16.5 | 131.8 | 269.8 | 41.09 |  | 1979 | 469.14 | 2.15 | | 2 | |
| VIC | 25 | 17 | 8 | 227 | 818 | 9 | 4.53 |  | 6.60 |  | 27.2 | 0.0 | 15.4 | 159.9 | 113.1 | 0.01 |  | 725 | 106.13 | 1.76 | | 1 | |
| SAN | 35 | 21 | 13 | 991 | 634 | 25 | 0.65 |  | 4.60 |  | 28.04 | 0.1 | 14.7 | 150.7 | 352.9 | 4.30 |  | 1392 | 205.61 | 1.85 | | 2 | |

**Island codes:** for the Azorean archipelago, COR (Corvo), FLO (Flores), FAI (Faial), PIC (Pico), GRA (Graciosa), SJO (S. Jorge), TER (Terceira), SMI (S. Miguel), SMA (Sta. Maria); for the Canary archipelago, FUE (Fuerteventura), CAN (Gran Canaria), GOM (La Gomera), HIE (El Hierro), LAN (Lanzarote), PAL (La Palma), TEN (Tenerife); for the Madeiran archipelago, MAD (Madeira), DES (Desertas), PSA (Posto Santo); for the Cape Verde archipelago, NIC (S. Nicolau), FOG (Fogo), ANT (S. Antão), VIC (S. Vicente), SAN (Santiago).

**Variable codes and data sources**

*A*: planar area^1^.

*D_M_*: distance to the closest mainland^1^.

*D_I_*: distance to the closest island^1^.

*N*: neighbor index^2^ [1]. It is calculated as:

$N=\sum_{i=1}^{24} \frac{A}{(D+1)^{2}}$, where *A* is the island area and *D* is the distance to the closest island.

*T*: oldest geological age estimated from the exposed aerial part of the island^3^.

*T_MAX_*: maximum temperature of warmest month^4^.

*P_MIN_*: precipitation of driest quarter^4^.

*T_S_*: temperature seasonality^4^.

*P_S_*: precipitatioin seasonality^4^.

*P_ANN_*: annual precipitation^4^.

*MistL*: orographic mist layer^5^.

*ELEV*: maximum altitude^6^.

*sdELEV*: standard deviation of altitude^6^.

*SLOPEdiv*: slope diversity^6^.

*EZ*: number of main ecological zones^7^.

^1^Most geographical variables (*A*, *D_M_*, *D_I_*) were obtained from Cardoso et al. [2] and GIS layers in the case of Cape Verde.

^2^The neighbor index (*N*) was calculated following Kalmar and Currie [1] as:

$N=\sum_{i=1}^{24} \frac{A}{(D+1)^{2}}$, where *A* is the island area and *D* is the distance to the closest island.

^3^The oldest geological age or time (*T*) for each island was obtained mainly from Triantis et al. [3], but also from Duprat et al. [4], Holm et al. [5] and Olehowski et al. [6].

^4^Climatic variables (*T_MAX_*, *P_MIN_*, *T_S_*, *P_S_*, *P_ANN_*) were obtained from WorldClim at 1 km resolution (data generated by interpolation of local stations for the 1960–1990 period; available at www.worldclim.org); values per island were calculated as the average of all island pixels.

^5^Orographic mist layer (*MistL*) was derived using the methodology proposed by Azevedo et al. [7], [8] which relies on the ability of the islands to promote and intercept orographic clouds from the orographically-induced adiabatic cooling of the air as it goes up the island in the context of a large scale advection. *MistL* can then be obtained by establishing the average elevation where dew point occurs (i.e. the ceiling of orographic clouds) and the ratio between the area above that level and the total area of the island.

^6^Topographical variables (*ELEV*, *sdELEV*, *SLOPEdiv*) were derived from a 90 m resolution digital elevation model (available at <http://srtm.csi.cgiar.org/>). Diversity of slopes (*SLOPEdiv*) was calculated as the Shannon diversity index of all pixel values.

^7^The number of ecological zones followed the classification proposed by Patiño et al. [9], and references therein.

**References**

1. Kalmar A, Currie DJ (2006) A global model of island biogeography. Global Ecol Biogeogr 15: 72-81.

2. Cardoso P, Arnedo MA, Triantis KA, Borges PAV (2010) Drivers of diversity in Macaronesian spiders and the role of species extinctions. J Biogeogr 37: 1034-1046.

3. Triantis KA, Borges PAV, Hortal J, Whittaker RJ (2010) The Macaronesian province: patterns of species richness and endemism of arthropods. In: Serrano ARM, Borges PAV, Boeiro M, Oromí P, editors. Terrestrial arthropods of Macaronesia. Lisbon: Sociedade Portuguesa de Entomologia. pp. 49-71.

4. Duprat HI, Friis J, Holm PM, Grandvuinet T, Sorensen RV (2007) The volcanic and geochemical development of Sao Nicolau, Cape Verde Islands: Constraints from field and Ar-40/Ar-39 evidence. Journal of Volcanology and Geothermal Research 162: 1-19.

5. Holm PM, Grandvuinet T, Friis J, Wilson JR, Barker AK, et al. (2008) An Ar-40-Ar-39 study of the Cape Verde hot spot: Temporal evolution in a semistationary plate environment. Journal of Geophysical Research-Solid Earth 113.

6. Olehowski C, Naumann S, Fischer D, Siegmund A (2008) Geo-ecological spatial pattern analysis of the island of Fogo (Cape Verde). Global Planet Change 64: 188-197.

7. Azevedo EB, Pereira LS, Itier B. Modeling the local climate in islands environments. Orographic clouds cover. In: Schmenauer RS, Bridman, editors; 1998; Ottawa, Canada. pp. 433-436.

8. Azevedo EB, Pereira LS, Itier B (1999) Modelling the local climate in island environments: water balance applications. Agric Water Manage 40: 393-403.

9. Patiño J, Carine MA, Fernández-Palacios JM, Otto R, Schaefer H, et al. (2014) The anagenetic world of spore-producing plants. New Phytol: 305-311.
