# Supplementary material for: Geographical, Temporal and Environmental Determinants of Bryophyte Species Richness in the Macaronesian Islands
Source: PLoS One. 2014 Jul 8;9(7):e101786. doi: 10.1371/journal.pone.0101786 (PMC4086965; doi:10.1371/journal.pone.0101786)
Supplement: File S3 — Spatial autocorrelation of model residuals and spatial autoregressive regressions (SAR) alternative to typical ordinary least squares (OLS) regression models. (DOCX) [file pone.0101786.s003.docx]

**Supporting Information S3.** Spatial autocorrelation of model residuals (see Fig. 1 and Table 2 in the main text), and spatial autoregressive regressions (SAR) alternative to typical ordinary least squares (OLS) regression models.

**Table S3.1.** Spatial autocorrelation of the residuals of the best models explaining moss species richness. The statistical significance of the Moran’s *I* coefficient was estimated using Monte Carlo permutations. It is shown the centroid of each distance class (km).

|  | GDM |  |  | HD |  |  | CLIMATE |  |
| --- | --- | --- | --- | --- | --- | --- | --- | --- |
| Distance | Moran’s *I* | *P* |  | Moran’s *I* | *P* |  | Moran’s *I* | *P* |
| 93. 7 | -0.39 | 0.005 |  | -0.12 | 0.623 |  | -0.13 | 0.503 |
| 248.1 | 0.02 | 0.809 |  | 0.13 | 0.377 |  | -0.04 | 0.829 |
| 440.8 | 0.07 | 0.693 |  | 0.17 | 0.307 |  | 0.05 | 0.834 |
| 889.9 | -0.20 | 0.171 |  | -0.24 | 0.161 |  | -0.27 | 0.116 |
| 1348.4 | 0.11 | 0.513 |  | -0.14 | 0.387 |  | -0.12 | 0.533 |
| 1571.5 | 0.02 | 0.874 |  | -0.12 | 0.397 |  | 0.06 | 0.643 |
| 1830.3 | 0.00 | 0.995 |  | 0.03 | 0.799 |  | 0.13 | 0.397 |

**Table S3.2.** Spatial autocorrelation of the residuals of the best models explaining liverwort species richness. Further details as in Table S3.1.

|  | GDM | |  | HD | |  | CLIMATE | |
| --- | --- | --- | --- | --- | --- | --- | --- | --- |
| Distance | Moran’s *I* | *P* |  | Moran’s *I* | *P* |  | Moran’s *I* | *P* |
| 93. 7 | -0.24 | 0.270 |  | 0.19 | 0.382 |  | -0.08 | 0.724 |
| 248.1 | 0.09 | 0.310 |  | 0.45 | 0.055 |  | 0.14 | 0.422 |
| 440.8 | 0.06 | 0.454 |  | 0.25 | 0.186 |  | -0.20 | 0.231 |
| 889.9 | -0.04 | 0.902 |  | 0.03 | 0.834 |  | -0.50 | 0.030 |
| 1348.4 | -0.13 | 0.590 |  | -0.34 | 0.075 |  | 0.03 | 0.834 |
| 1571.5 | -0.16 | 0.417 |  | -0.59 | 0.010 |  | 0.28 | 0.131 |
| 1830.3 | 0.05 | <0.001 |  | -0.25 | 0.116 |  | -0.05 | 0.683 |

**Table S3.3.** Goodness-of-fit of the spatial autoregressive regressions (SAR). It is shown the explained variance (*R^2^*) and the sample size-corrected Akaike information criterion (AIC*_C_*) for all the hypotheses. Cf. Table 2 in the main text.

|  | *F* | *P* | *R^2^* | AIC*_C_* |
| --- | --- | --- | --- | --- |
| **Moss species richness (*S_M_*)** |  |  |  |  |
| GDM (*A*, *TT*^2^) | 10.54 | <0.001 | 0.676 | 217.5 |
| HD (*sdELEV*) | 26.13 | <0.001 | 0.599 | 213.4 |
| CLIMATE (*P_MIN_, MistL*) | 17.03 | <0.001 | 0.683 | 212.7 |
| **Liverwort species richness (*S_L_*)** |  |  |  |  |
| GDM (*A*, *TT*^2^) | 6.00 | 0.011 | 0.456 | 197.8 |
| HD (*sdELEV*) | 5.41 | 0.033 | 0.257 | 200.0 |
| CLIMATE (*T_MAX_, P_MIN_, MistL*) | 19.22 | <0.001 | 0.823 | 180.9 |
